# Unilateral Facial Paralysis in an Infant Post-vaccination: Insights Into Bell’s Palsy

**DOI:** 10.7759/cureus.79971

**Published:** 2025-03-03

**Authors:** Sonal Kumar, Adnan A Islam, Patricia Ward, Taylor E Collignon, Adriana Castro

**Affiliations:** 1 Surgery, Ross University School of Medicine, Miramar, USA; 2 Neurology, Ross University School of Medicine, Miramar, USA; 3 Internal Medicine, St. George's University School of Medicine, St. George's, GRD; 4 Internal Medicine, Lake Erie College of Osteopathic Medicine, Bradenton, USA; 5 Pediatric Medicine, Castro Pediatrics, Miami, USA

**Keywords:** childhood immunization, facial nerve paralysis, immune-mediated neuropathy, infant neurology, neuroinflammation and vaccination, pediatric bell’s palsy, pediatric neurology, post-vaccination complications, seventh cranial nerve palsy, vaccination adverse events

## Abstract

Facial nerve palsy is already a rare adverse effect in infants, and its association with routine vaccinations is even less common. Our case report shares the story of a unique instance of Bell’s palsy in a two-month-old infant with unilateral facial paralysis one day following standard immunizations. Neuroimaging revealed enhancement of the left seventh cranial nerve, consistent with Bell’s palsy with lower motor neuron involvement. An extensive infectious workup, including polymerase chain reaction (PCR) and serologies for a wide range of pathogens, was negative, and cerebrospinal fluid analysis indicated no signs of infection or inflammation. Given the temporal association with vaccination, our clinical case raises important questions about the potential neurological side effects of immunizations in very young infants. While this condition typically has an encouraging prognosis, there exists a significant gap in research regarding the pathogenesis and etiology of Bell’s palsy as it relates to pediatric vaccination. Our report highlights the need for further investigation into the risk of post-vaccination neurological complications, particularly in the pediatric population.

## Introduction

Facial nerve palsy (FNP) is a rare condition in infancy, though it can occasionally occur as a neurological sequelae following routine vaccinations. The annual incidence of FNP in the pediatric population under 10 years of age is approximately 2.7 per 100,000, with an even lower incidence presumed in infants [1]. In children, FNP is typically classified as either congenital-often due to birth trauma or complications from assisted vaginal deliveries-or acquired, with a broad range of potential causes including infections, inflammation, neoplasms, and iatrogenic factors. Interestingly, some cases have been linked to autosomal dominant inheritance with incomplete penetrance [2].

The most common cause of unilateral facial paralysis in children is idiopathic, known as Bell’s palsy, which is frequently associated with viral infections. While Bell’s palsy has been linked to viral reactivation, there is no evidence suggesting that the routine vaccines given at the two-month immunization visit-such as hepatitis B; diphtheria, tetanus, and pertussis (DTaP); *Haemophilus influenzae* type b (Hib); inactivated poliovirus; and pneumococcal conjugate vaccine (PCV13)-are live-attenuated vaccines that could trigger viral reactivation [3].

Treatment of Bell’s palsy depends on the underlying cause. For acquired cases, corticosteroids are often used and have been shown to improve outcomes in pediatric patients, while antiviral therapy may be indicated when a viral cause is suspected. In more severe cases, surgical interventions such as facial reanimation surgery may be necessary [4]. Despite the generally positive prognosis for FNP in children, there remains a significant gap in research regarding the pathogenesis and potential links between Bell’s palsy and the two-month vaccination series.

This case presents a rare instance of Bell’s palsy occurring in a two-month-old infant one day after routine immunizations, highlighting the need for further investigation into the possible neurological complications associated with vaccines.

## Case presentation

An otherwise healthy four-month-old female presented to her pediatrician after a sudden-onset left-sided facial droop for which she went to the hospital. The droop began after receiving her two-month vaccination series, which included Prevnar 13, Pentacel (DTaP-inactivated poliovirus vaccine (IPV)/Hib), and rotavirus. The patient’s mother also confirmed the patient had an upper respiratory infection (URI) one week prior to hospital admission, including nasal congestion and rhinorrhea.

During hospitalization, the infant also had a rash on the left upper extremity. She ultimately received a diagnosis of impetigo, which was treated with Mupirocin 2% ointment for two days. Per mom, the patient did not have fever, irritability, or other systemic symptoms. A comprehensive workup was conducted during hospitalization. A lumbar puncture and cerebrospinal fluid analysis both showed no signs of infection or inflammation. Infectious workups including polymerase chain reaction (PCR) and serologies for a wide range of pathogens including herpes simplex virus (HSV), *Mycoplasma pneumoniae*, Epstein-Barr virus (EBV), varicella-zoster virus (VZV), *Enterovirus*, adenovirus, SARS-CoV-2, respiratory syncytial virus (RSV), *Bordetella pertussis*, chlamydia, parainfluenza virus, and influenza virus were negative. Additionally, laboratory tests revealed mildly elevated transaminases (Table 1). The patient was diagnosed with Bell’s palsy and started on a seven-day tapering course of oral prednisolone (15 mg/5 mL solution). Impetigo resolved with topical Mupirocin.

**Table 1:** Summary of cerebrospinal fluid analysis and laboratory test results, including mildly elevated transaminases Hct: hematocrit; Hgb: hemoglobin; MCH: mean corpuscular hemoglobin; MCHC: mean corpuscular hemoglobin concentration; MCV: mean corpuscular volume; MPV: mean platelet volume; RDW: red cell distribution width; BUN: blood urea nitrogen; ALT: alanine aminotransferase; AST: aspartate aminotransferase; Alk Phos: alkaline phosphatase

Laboratory test	Result	Reference
Hct	35.1%	29.0-42.0
Hgb	12.1 mg/dL	10.0-14.5
MCH	28.6 pg	27.0-33.0
MCHC	34.5 mg/dL	32.0-36.0
MCV	83.0 fL	74.0-108.0
MPV	9.9 fL	7.4-10.4
RDW	12.0%	11.5-14.5
Platelet	467 × 10 K/μL	242-378
Basophil	0.0 × 10 K/μL	0.0-0.1
Eosinophil	0.4 × 10 K/μL	0.0-0.8
Lymphocytes	5.4 × 10 K/μL	2.3-14.4
Monocytes	1.6 × 10 K/μL	0.0-0.5
Neutrophil	7.2 × 10 K/μL	1.0-8.5
Creatinine	<0.15 mg/dL	0.30-0.60
BUN	4 mg/dL	5-27
CO_2_	16.7 mmol/L	16.0-28.0
Glucose	87 mg/dL	70-123
Calcium	9.8 mg/dL	9.0-10.9
Sodium	136 mmol/L	131-145
Potassium	5.6 mmol/L	3.9-6.4
Chloride	104 mmol/L	98-118
ALT	42 IU/L	12-37
AST	73 IU/L	20-63
Total protein	7.0 g/dL	4.3-6.9
Albumin	4.7 mg/dL	2.7-4.8
Bilirubin total	1.3 mg/dL	0.2-1.3
Alk Phos	138 IU/L	80-345

Of note, the pregnancy was complicated by maternal anemia for which the mother had four iron infusions. The infant was delivered full term via an uncomplicated spontaneous vaginal delivery. The mother also reported positive for Pap smear for human papillomavirus (HPV) approximately one month before the infant developed facial droop.

**Figure 1:**
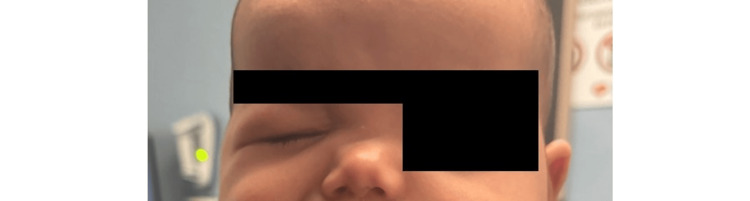
Image showing left-sided facial weakness and drooping of the patient's left eye

**Figure 2:**
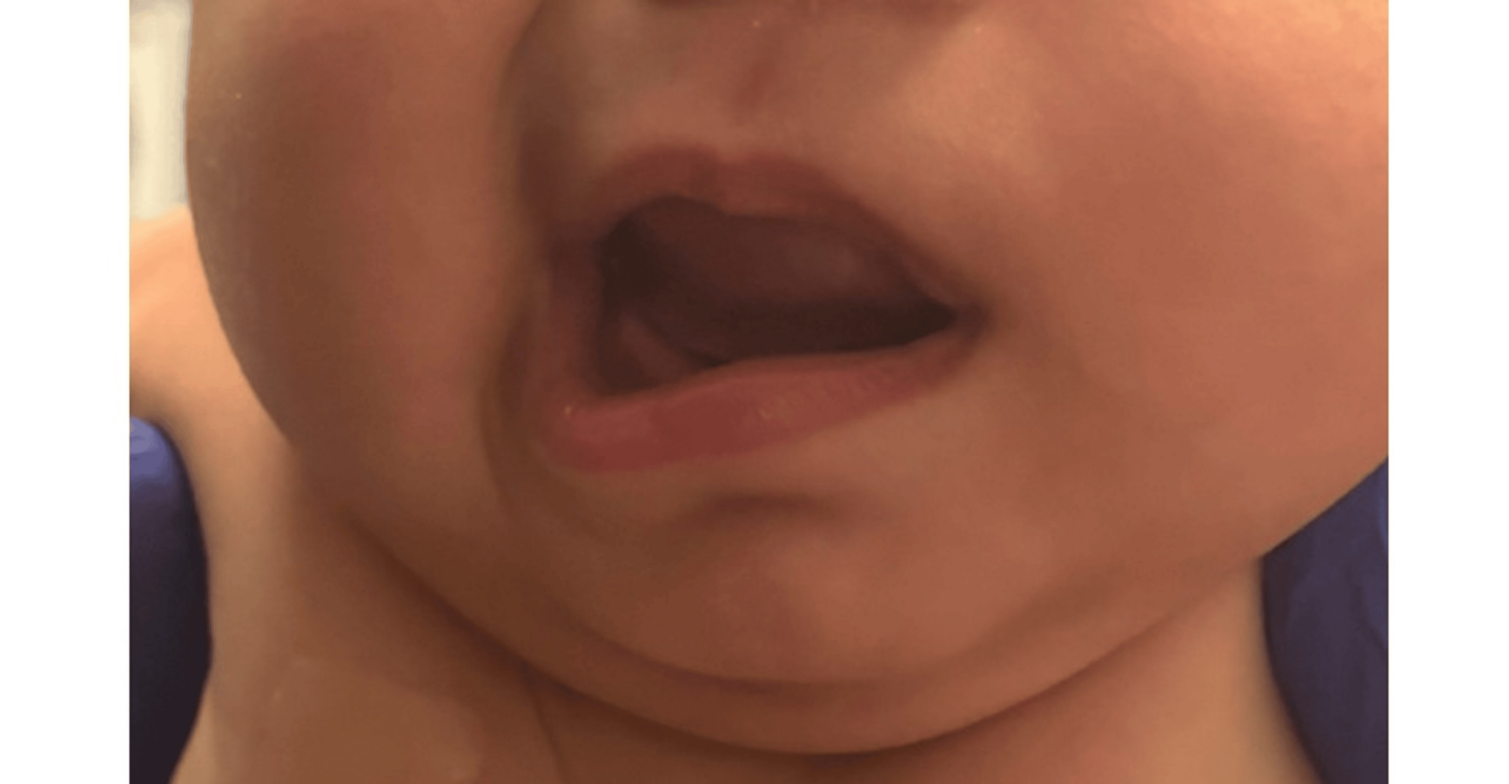
Image showing left-sided facial weakness and drooping of the left half of the patient's face

At the time of discharge, the patient demonstrated partial improvement in facial weakness. During an outpatient follow-up visit two weeks later, further improvement was noted, though mild residual weakness persisted (Figures 1, 2). This was most evident in the left eye and left lip, particularly when smiling. This case highlights a rare presentation of Bell’s palsy in an infant, potentially linked to preceding URI symptoms and temporally associated with routine vaccinations.

## Discussion

Bell’s palsy is an acute FNP associated mostly with viral infections, autoimmune conditions, and inflammatory processes. It commonly presents in adults. Pediatric presentations are, therefore, uncommon. While the pathophysiology of Bell’s palsy is unclear, reactivation of HSV in the geniculate ganglion is a leading hypothesis [5]. Triggers such as EBV, VZV, and immune-mediated mechanisms may also contribute [6].

Our case presentation is an important addition to the literature because we discuss the temporal relationship between the onset of Bell’s palsy and the administration of the infant’s routine two-month vaccinations. Bell’s palsy occurring following vaccination is reported in the literature, though it remains an uncommon event [7]. Literature has described cases of FNP following the administration of vaccines such as the COVID-19 vaccination [8], hepatitis B vaccine [9], and HPV vaccine [10]. These associations may suggest that an immune-mediated response is responsible. Still, however, more epidemiological studies are warranted as a causal link between vaccination and Bell’s palsy has not been established [11].

One proposed mechanism for post-vaccination Bell’s palsy includes an immune-mediated inflammatory reaction leading to facial nerve edema and compression in the facial canal [12]. Molecular mimicry where vaccine antigens trigger an immune response that cross-reacts with host neural tissues has also been proposed as another mechanism [12]. However, given the overall rarity of Bell’s palsy following immunization, the risk remains low compared to the benefits of vaccination in preventing serious infectious diseases.

Future studies such as large-scale surveillance data and population-based cohort studies are necessary to clarify such proposed associations. Clinicians must remain vigilant for post-vaccination neurological events yet still advocate for routine immunization given the overall public health benefits.

## Conclusions

Our patient represents an unusual case of FNP following immunizations. This unusual side effect raises the question of whether nerve palsy seen here is immune-mediated or inflammatory. The etiology of Bell’s palsy is idiopathic; the infant’s favorable outcome with corticosteroid therapy suggests favorable prognosis in pediatric patients. Given the rarity of FNP in infants, this case addresses the importance of post-vaccination side effects to spread awareness of uncommon events. We would like to emphasize the benefits of routine immunizations far outweigh the risks of adverse events. Additional research is necessary to understand the mechanisms involved. Clinicians should remain vigilant in identifying, reporting, and managing rare complications.
